# Collaboration over competition? Regulatory reform and inter-organisational relations in the NHS amidst the COVID-19 pandemic: a qualitative study

**DOI:** 10.1186/s12913-022-08059-2

**Published:** 2022-05-13

**Authors:** Justin Avery Aunger, Ross Millar, Anne Marie Rafferty, Russell Mannion

**Affiliations:** 1 School of Health Sciences, University of Surrey, Guildford, GU2 7YH UK; 2 Health Services Management Centre, Park House, University of Birmingham, Birmingham, B15 2RT UK; 3 Florence Nightingale Faculty of Nursing, Midwifery and Palliative Care, King’s College London, London, SE1 8WA UK

**Keywords:** Regulation, Policy, NHS, Collaboration, Competition, Integrated care, Integration, Healthcare, COVID-19

## Abstract

**Background:**

In 2021, during the COVID-19 pandemic, England’s Department of Health and Social Care (DHSC) released a White Paper outlining proposed legislative reform of the National Health Service (NHS). Key to the proposals is the shift from relationships between providers based on competition, to cooperation, as the central driver of improved performance and quality. Against this background we explore potential regulatory barriers and enablers to collaboration identified by key NHS stakeholders and assess whether the proposed policy changes are likely to deliver the desired improvement in collaborative relationships, in the context of challenges experienced during the COVID-19 pandemic.

**Methods:**

We conducted 32 semi-structured interviews with 30 key stakeholders, taking place during the COVID-19 pandemic from Jan 2020 to May 2021. Participants were selected for their expertise regarding collaboration and were recruited purposively. Interviews were conducted online with the use of video conferencing software. The interviews were thematically analysed to identify themes. Proposals contained in the DHSC White Paper helped to structure the thematic analysis, interpretation, and reporting of the results.

**Results:**

Requirements to compete to provide services, regulatory ability to block collaborative arrangements, lack of collaboration between providers and Clinical Commissioning Groups, and current lack of data sharing were found to hamper collaborative efforts. These issues often negatively affected collaborative relations by increasing bureaucracy and prompted leaders to attempt to avoid future collaborations. Other barriers included opaque accountability arrangements, and erosion of trust in regulators. The COVID-19 pandemic was found to foster collaboration between organisations, but some changes mandated by the new legislation may stifle further collaboration.

**Conclusions:**

Many of the proposed legislative changes in the White Paper would help to remove existing barriers to service integration and collaboration identified by stakeholders. However, the proposed shift in the concentration of power from NHS England to the DHSC may exacerbate historically low levels of trust between providers and regulators. Many of the proposed changes fail to address endemic NHS policy issues such as chronic understaffing. Further dialogue is needed at all levels of the health and social care system to ensure future legislative changes meet the needs of all stakeholders.

**Supplementary Information:**

The online version contains supplementary material available at 10.1186/s12913-022-08059-2.

## Introduction

Whether collaboration or competition is the best mechanism for driving improved performance in the healthcare system has long been debated in the United Kingdom [1, 2]. Indeed, over the last decade, the balance between competition and collaboration has shifted many times [3, 4]. For example, the Health and Social Care Act of 2012, also termed the ‘Lansley Reforms’ after its architect, former Secretary of State Andrew Lansley, introduced requirements for competition and competitive tendering in the NHS and created Clinical Commissioning Groups (CCGs) with the stated aim to improve the sensitivity of the commissioning system to the needs of patients [5, 6]. The 2012 act was widely viewed as increasing the risk of privatisation and imposing barriers to collaboration, while also handing over more power to NHS England [7]. However, as soon as 2014, regulators began mandating some forms of collaboration between providers, including buddying and mergers, as a means of turning around poor quality care in response to poor inspection results [8].

During this time, organisations were often simultaneously competing in the market while also engaging in collaborative relationships (e.g. involved in ‘buddying’ a Trust while engaging with competitive commissioning), demonstrating that collaboration and competition are not always an ‘either or’ scenario [9]. Similarly, in the USA, hospitals often engage in interorganisational collaboration to deliver a range of services, while competing for government funding, patients, and healthcare professionals [10]. The simultaneity of a high degree of both competition and collaboration in a health system has been labelled as ‘coopetition’ [10]. Zhu [10] highlights that while competition can enhance the need for innovation, interorganisational collaboration can reduce risk when innovating, expedite information sharing which is key to such breakthroughs, and spread innovation. Despite competition and collaboration both fostering innovation through different mechanisms, evidence suggests that, given the choice, most NHS leaders prefer collaborating over competing [3].

Since collaboration was encouraged in the Five Year Forward View of 2014, as well as in the Dalton Review [11], the NHS embarked on testing several ‘new care models’ (also termed ‘Vanguards’) in a number of locations to assess how providers from acute, social, and primary care are able to work together to improve financial viability and reduce variation in care [11–13] (Table 1). This was also in response to the Carter Report of 2016, which suggested that £5 billion of cost savings could be made through reducing unwarranted variation in acute service provision [15]. Since the Vanguards started being tested, certain aspects of the rules designed to retain competition between providers have largely fallen into abeyance [3]. Likewise, from the perspective of healthcare providers, the use of incentives by central authorities to compete in some areas, and collaborate in others, has led to much confusion in the provision and commissioning of services [3]. In some cases it is evident that many senior leaders in the NHS are not fully aware of the current regulations, with some commentators noting that “actors need to understand the rules of the game” [3]. The uncertainty around the regulatory environment may also discourage NHS leaders from engaging fully with a collaborative agenda until suitable legal frameworks are in place [3].

**Table 1:** Explanation of key concepts

Concept	Definition
National Health Service (NHS)	The National Health Service of the United Kingdom. It is a publicly-funded service that provides universal healthcare and is free at point of use.
Department for Health and Social Care (DHSC)	The DHSC is the government branch responsible for health and social care policy in the United Kingdom, with a primary focus on England.
2012 - Health and Social Care Act	This 2012 Act introduced requirements for competition and competitive tendering in the NHS and created Clinical Commissioning Groups (CCGs) with the stated aim to improve the sensitivity of the commissioning system to the needs of patients. It also introduced other requirements for competition such as the role and ability for Monitor to ensure that any collaborative organisational entities would require review for potential anti-competitive practices and would block proposals if required.
2014 - NHS Five Year Forward View	Published in October 2014, this report set out the plan for the NHS in England for the next five years. This outlined the move towards more collaborative structures such as Multispecialty Community Providers and Primary and Acute Care Systems and was the initial divergence from the 2012 Health and Social Care act only a couple years prior.
2021 - DHSC White Paper “Integration and innovation: working together to improve health and social care for all”	A White Paper released in early 2021 which set out legislative proposals for a future Health and Social Care Bill, setting the future direction for the health system in England.
2022 - Health and Care Bill	The legislative means for achieving what was set out in the DHSC White Paper, to be enacted in 2022 (see Table 2).
Collaboration (inter-organisational)	Organisations coming together with the intention to achieve benefits that they would not be able to achieve alone.
Integration	A form of collaboration which generally results in subsummation of one organisation into the umbrella of another.
Integrated care	Usually refers to horizontal integration, which is when providers of different health services (e.g., mental health and acute care) are brought together.
Competition (in the NHS)	Refers to the use of a market system in the NHS, which was introduced in the 1990s, and a split between provision and commissioning of services intended to drive improved patient choice.. In 2012, this also involved the implementation of anti-competitive laws that could prevent certain collaborative organisational behaviour deemed to limit patient choice as well as collaborations between providers and commissioners that could be seen as unfair. This has also led to a focus on individual organisational performance that might come at the expense of local system performance.
Coopetition	A term used in the organisational science literature to describe an environment whereby “*dense collaborative relationships exist in highly competitive markets*” [10].
Clinical Commissioning Groups (CCGs)	Introduced in 2012, these are groups of general practices that come together to buy services for their patients and population.
NHS Vanguards	Tests of five different new models of care that were piloted in England in 2015–2018 after which many continued without additional funding in place. The focus was, in most cases, to improve the care pathway through horizontal integration.
Sustainability and Transformation Partnerships	Precursors to Integrated Care Systems, these were introduced in NHS planning guidance in late 2015 and sought to lead to ‘place-based planning’ with the NHS and social care system working more closely together to better manage collective resources. This involved separating England into 44 ‘plan areas’ with leaders appointed for each that were to implement the Five Year Forward View. Many of these have since transformed into Integrated Care Systems.
Integrated Care Systems	New forms of collaboration that seek to build upon the 2022 Health and Care bill to horizontally integrate services as well as bring together providers and commissioners in a way that was not possible under prior competitive law. There are 42 ICSs currently implemented.
Care Quality Commission (CQC)	A public body within the DHSC, this body regulates and inspects health and social care services in England to ensure they provide safe and high quality care.

To address these issues, a new Health and Care bill was introduced by the Department of Health and Social Care (DHSC) in early 2021 and is, as of early 2022, currently being considered in the House of Lords [16]. This bill signals the need to shift from competition to collaboration in planning and commissioning by 2022 [17].“Integration is the new competition” is the summary message from the bill [18]. The COVID-19 pandemic features heavily in the White Paper, acknowledging the changes that have been made to ease collaboration to tackle the pandemic, going so far as to state that “﻿*we must not go back to the old ways of working*” and that the “﻿*gains made through these new approaches must be locked in*”. It also argues that “*COVID-19 has demonstrated the importance of different parts of the health and care system working together in the best interests of the public and patients* … ﻿*despite the barriers in legislation which sometimes make it difficult to do so*”. Such changes made during the COVID-19 pandemic included, for example, enabling the NHS Commissioning Board to bypass CCGs to buy services directly from the private sector as well as more general flexible takeovers of tasks from CCGs by NHS England [19]. It is important to explore how these changes impacted on the ability of key stakeholders to collaborate.

To ‘lock in’ these developments and move the system towards greater collaboration, the White Paper proposes giving the NHS and local authorities a duty to collaborate, making Integrated Care Systems (ICSs) statutory bodies, reducing bureaucracy, among many other changes (Table 2). ICSs are the means for accomplishing much of this agenda, constituting multiple partnerships between organisations including local council, community, and voluntary organisations, intended to enhance place-based care. These new partnership arrangements have been mandated to come into force in April 2021 as part of the NHS Long Term Plan of 2019, building upon prior Sustainability and Transformation Partnership (STP) and Vanguard arrangements. However, the statutory implementation and closure of existing CCGs has been delayed to July 2022 [20].

**Table 2:** Summary of key proposed changes relating to collaboration outlined in the 2022 Health and Care Bill

1. Providing a duty for the NHS and local authorities to collaborate with the Triple Aim (improving patient experience, reducing per-capita healthcare cost, and improving population health) as a focus [14]	
2. Making ICS’ statutory bodies, comprised of an ICS Health and Care Partnerships (bringing systems together to support integration) and an ICS NHS Body (responsible for day-to-day running of the ICS) 3. Enshrining commissioning in the ICS NHS Body • The current role of clinical commissioning groups (CCGs) will be taken over by the ICS NHS Body to enhance accountability and strategic planning ability. • Enabling NHS providers and CCGs (now ICS NHS Bodies) to *legally* take joint decisions with use of joint committees and committees-in-common arrangements, as well as bring in other partners, e.g., GP practices, voluntary sector • Allowing groups of ICSs to use joint commissioning to deliver combined services	
4. Reducing bureaucracy by: • Removing competition law, the Competition and Markets Authority (CMA), as well as NHS Improvement competition features and anti-competition duty • Eliminating the need for competitive tendering if not providing value • Reforming the National tariff system towards collaboration and a focus on population health • Removing the requirement for Local Education and Training Boards • Giving the Secretary of State the power to create new Trusts as required to enhance ICS delivery	
5. Improving accountability by: • Merging NHS England and NHS Improvement • Shifting accountability from NHS England into ICSs themselves with oversight from the Secretary of State • Ensuring a more agile and flexible framework for national bodies • Enacting legislation to improve social care accountability	
6. Enhancing governmental powers of direction over newly merged NHS England body	
7. Allowing joint appointments of executive directors across NHS Bodies, local authorities, and Combined Authorities, and a combination thereof	
8. Improving data sharing across the system	

The proposals outlined in the White Paper moves away from many aspects of the Coalition Government’s previous agenda set out in the 2012 Health and Social Care Act. They could be considered a retreat from many aspects that have not worked as well as originally intended [21]. This prior Act emphasized competition as the primary strategy to achieve efficiency and quality of service. The new legislation has, on the one hand, been praised by some for enabling a shift towards greater collaboration - as has been widely acknowledged as being required to deal with the pressures of the COVID-19 pandemic - while, on the other, has prompted warnings about handing power from NHS England to the Secretary of State for Health and Social Care [21]. Some critics argue that devolution and vesting power in local systems, where such decisions are perhaps better made, may improve outcomes than changing than centralising decision-making [5]. Likewise, The King’s Fund (2021), has warned that “*the government and national NHS leaders should be looking to step away from the damaging model of top-down command and control in the NHS”.*

Other critics argue that the tensions within the healthcare system in recent years mean the timing of the proposed changes is not optimal. There have been concerns that the NHS is under immense strain and does not currently have the capacity to implement a wide-ranging reorganisation [22, 23]. In addition, questions have been raised as to why the White Paper only briefly mentions the key underlying issues facing the NHS, including the need to address chronic staff shortages and widening health inequalities [7, 18]. Indeed, there is insufficient evidence to support the view that inter-organisational collaboration actually leads to the intended improvements in outcomes, while being incredibly difficult to achieve [24–27]. Thus, collaboration may not be a ‘magic bullet’ to solve the health system’s ills.

Against this background, we sought insight from NHS leaders, policymakers, and patients, regarding how regulation since the 2012 Health and Social Care Act has affected the implementation of collaborative and inter-organisational arrangements in the NHS. We also sought to analyse how the policy changes outlined in the White Paper may resolve issues identified by our interviewees. Finally, we sought to explore how the COVID-19 pandemic has affected inter-organisational collaboration in the English NHS in the context of this legislative uncertainty.

## Methods

### Participant selection

A purposive convenience sample recruited NHS leaders, frontline staff, and those working at regulators and commissioners in England for interview, as part of a larger project seeking to understand how and why inter-organisational collaborations in healthcare work [26, 28]. As such, these stakeholders could be seen as members of a wider ‘issue network’ related to collaboration in the NHS [29]. All interviewees had direct, relevant experience of either working on policies related to implementation of inter-organisational collaborations in the NHS directly, or working as part of an inter-sectoral collaboration (e.g., in the charity sector). We endeavoured, where possible, to gain representation, particularly in the case studies, of both senior and junior staff. However, the pandemic made it difficult to gain access to interview frontline staff. All interviews took place in the context of the NHS Long Term plan and during the COVID-19 pandemic. Due to practical difficulties associated with the pandemic, our sample was mostly obtained through networks linked to our advisory group for the study, as well as through snowballing from initial contacts. Therefore, in practice, we used a purposive convenience sample with snowball elements. Participants were approached to participate via email.

Our final sample comprised 32 interviews with 30 stakeholders. These were conducted between January 2020 and May 2021. Interviews were with Executives, senior leaders of collaboration (e.g., Head Nurses) (*n* = 18) who have experience with collaboration in the NHS to date and will be affected by these system-wide changes, and policymakers from NHS England/Improvement, commissioners, regulators, and other organisations (*n* = 12) Table 3 outlines the characteristics of the participants. All interviews included coded excerpts regarding the impact of regulation on collaboration.

**Table 3:** Overview of stakeholder interviews

Case studies of IOC programmes	Role (Interview code)
Hospital Group 1 (South)	Director for strategy (2) × 2
Hospital Group 2 (South)	Director of partnerships (3) × 2
Hospital Group 3 (South)	CEO of hospital group (18)
Hospital Group 4 (South)	Lead of alliance organisational design (29) Director of Improvement (30)
Alliance 1 (North)	Executive Nurse & deputy CEO (10)
	Former CEO and architect of alliance model (12)
	Delivery Officer (20)
	Current CEO of Alliance (22)
	Medical Director for Committees in Common (23)
	Workforce Director of HR in the Alliance (26)
Alliance 2 (North)	CEO of a trust in the alliance (17)
	Director of the overall alliance (19)
ICS 1 (North)	CEO of partnership (13)
ICS 2 (South)	ICS Lead (14)
ICS 3 (South)	CEO of the ICS (25)
Integrated Care Provider (North)	Commissioning lead for the partnership (16)
Merger (South)	Director of clinical service being merged (21)
**Wider stakeholder perspectives**	
Academic with partnership expertise & Non-Exec of a hospital group (1)	
Provider Policy Lead at key national NHS body (4)	
Provider Policy Inspectorate Strategy Lead (5)	
NHS Provider Association Policy and Strategy Advisor and Director (6 (× 2) and 11)	
Professional Regulatory Body CEO (7)	
Regional Hospital Inspectorate Lead for National Inspectorate Body (8)	
Senior Advisor on systems transformation at National Body (9)	
Patient Representative Lead at Health and Social Care champion body (15)	
Director of Third Sector/Charity Representative (24)	
Local Government Association Representative (28)	
Private Sector Representative with experience of private/public partnership (27)	

### Interviews and setting

Interviews were conducted by experienced qualitative interviewers (JA, R Millar, and AMR). The interviews were semi-structured and formed part of a larger realist evaluation project [26, 28, 30]. The interview guide is available in supplementary file 1, but this evolved throughout the project, and we often followed leads as they arose in the interviews. Due to the impact of the pandemic shortly after embarking on the research, interviews were conducted virtually over Zoom or Microsoft Teams and recorded on an external, dedicated, encrypted audio recorder. Interviews lasted between 30 and 90 minutes but were typically closer to 60 minutes in length. Files were sent for verbatim transcription at a third-party transcription service.

### Theoretical framework and data analysis

Themes in the Department of Health and Social Care’s (DHSC) policy White Paper, “Integration and Innovation: Working together to improve health and social care for all” was used to guide the coding process and for the writing of this study report [17]. Inductive thematic analysis was performed with the aid of NVivo 12 software by one coder (JA) with coding logic independently verified by a secondary coder (R Millar). This meant that as transcripts were read, new codes were created as required, and sub-codes made for any recurring themes that fit within larger criteria (e.g., ‘requirement to compete’). This article is written according to the Standards for Reporting Qualitative Research (SRQR) [31].

## Findings

Our qualitative analysis was structured around issues and themes outlined in the White Paper. Our analysis focuses primarily on the context for and potential impacts of themes including the duty to collaborate, reducing bureaucracy, improving accountability, enhancing governmental powers of direction, and improving data sharing across the system. Additionally, we include an analysis of how the process of collaboration was impacted by the COVID-19 pandemic and policy changes which occurred during the pandemic. These themes are elaborated on below.

### Implementing a ‘duty to collaborate’ without disrupting existing collaborative structures

The overall sentiment in our interviews was that regulatory reform was required for the effective implementation of ICS’ and other forms of horizontal collaboration such as provider collaboratives. The overall sentiment about the current market system was put forward by one NHS Leader, noting that:

*“ … the law hasn’t changed so they’re set up on the basis of competition [ … ] because of the way it operates it undermines, actually, the quality and investment that can be made in services on an ongoing basis, and I think that’s a real sharp end factor in terms of the competition piece.“* (11; Policy executive)

However, the pace of change also needed to be managed. One interviewee, who was head of partnerships at an ICS, noted how they were still nurturing all the relationships in their collaboration, and that everyone was vying for a position on the NHS body, where it was not possible for everyone to sit.

“ … *If anything, we could have had another good couple of years without the White Paper. And so, it helps to solidify certain things I just need to navigate this quite carefully with them all so they don’t lose their momentum and their commitment.”* (25; Lead; ICS 3)

This made clear that the White Paper has the potential to disrupt the balance of relationships that had been nurtured prior to the proposed changes, many of which are based upon fragile political structures. This view was echoed by an interviewee who was critical of this situation:

“*And then there’s the dead hen, or the bureaucracy that says, “Oh, we want you to work all collaboratively,” and what’s happening is they’re producing volume after volume of telling us how to work collaboratively. And they’re going to assess us against frameworks about it, and you think … [ … ] Well I don’t know that for certain, but there’s a strong rumour. I do know for certain there is a framework for assessment of the maturity of your collaboration as acutes. I’m thinking, “That is bonkers.*” (22; CEO; Alliance 1)

### Reducing bureaucracy

The 2012 Act and Lansley reforms were widely criticised for increasing bureaucracy, and this problem is reflected in reforms set out in the White Paper [17]. One of the central aims of the proposed legislation is ‘reducing bureaucracy’ and placing “pragmatism at the heart of the system”. This is to be achieved through a combination of modifications, including changes to competition law, procurement rules, and reforms to the tariff. In our study, bureaucracy was often cited as a barrier to engaging in collaboration. In the past, the CMA viewed as very bureaucratic, with providers having to rationalise their proposals in very burdensome business cases which often took years to deliver. One leader summarised one example of the problems they faced with bureaucracy as follows:

*“We had to do a full business case to acquire the other three parts of [Acute hospital], and demonstrate what the benefits were of us acquiring it, and what improvements that organisation would see [ … ] “What are the benefits of us giving you this organisation?” “Well actually, you gave it us three years ago, sort yourselves out. This is ridiculous.”* (10; Exec Nurse; Alliance 1).

However, in recent years, the CMA has played a much-reduced role in approvals of such cases. Yet, this demonstrates that its legacy may continue to dissuade leaders who have been around for many years from engaging collaboratively with other organisations.

#### The impact of competition law and competition and markets Authority (CMA)

The CMA has historically presented a barrier to the merger of foundation trusts by providing additional bureaucracy as well as real potential for actual blockages of collaborative efforts [17, 32, 33]. However, in reality, the CMA has been relatively absent in the health market since a the landmark decision in the merger of Central Manchester University NHS Foundation Trust and University Hospital of South Manchester NHS Foundation Trust in 2017 [34]. This decision made it clear that the CMA was moving the ‘competition test’ away from assessing whether providers deliver the same services to a patient benefit focus [32]. The shift in emphasis from competition to collaboration since the NHS Five Year Forward View of 2014 had not gone unnoticed by leader of provider organisations:

*“If competition was the kind of policy kind of organising instrument, in the kind of period 2010 to 2015 … the Lansley Reforms, maybe even more recent than that, then certainly in the last couple of years have been … okay, that competition thing, we’re dialling that down, it’s all about collaboration.”* (04; Provider Policy Leader)

There was also a pervading sense that there have been significant moves within the healthcare system towards forms of collaboration that are necessary for the effective delivery of services regardless of the regulatory structure, particularly since the publication of the NHS Long Term Plan [35] and during the COVID-19 pandemic. One policymaker outlined this as follows:

*“There is another factor here which is again a kind of a sort of, you know, de facto/de jure issue I think which is de facto collaboration is happening, de jure the framework isn’t there to enable it to work properly so [ … ] we’ve got ICSs and STPs across the country, they have no statutory underpinning whatsoever.”* (11; Policy Executive).

Nonetheless, competition rules still presented a perceived barrier to providers.. It was clear from our interviews that CMA rules and their ability to block mergers has guided even initial thoughts about what forms of collaboration may or may not be possible at leadership level. Another Chief Executive made it clear that choice of partner was still questioned due to apprehension regarding the role of the CMA, with organisations unwilling to collaborate with providers delivering similar services in local areas. This drove providers to join particular collaborative forms such as hospital groups, as an alternative to mergers, for example:

*“Now for the [large hospital group] so part of the reason that [large hospital group] was thought to be a good partner was that again there would be no CMA problem because they were in a different patch.”* (03; Director of Improvement; Hospital Group 2)

In some cases, these competition requirements disincentivised initiatives for engaging in collaborative activity between providers altogether. One inspectorate leader noted that:

“.. *it is a key policy system challenge that you’ve got the 2012 Act that’s pulling in one direction, we’ve got things pulling in the other direction*. *So, the straddling of the incentives to compete and all that goes with it, with the ideas to integrate and work – yeah, they’ve got to, we’ve got to … sort out some of the legal levers“* (08; Regional Inspectorate Lead).

The requirement from the CMA for organisations not to deliver the same services has led to providers pursuing vertical forms of integration, even where horizontal forms may been a better means of improvement [36]. This is reflected in the following quote by the interviewee, in which they also refer to the blocking by the CMA in 2013 of the merger of Bournemouth and Poole NHS Foundation Trusts. This was still preoccupying the decisions of leaders 7 years later:

*“So, what was fresh in our minds then was Bournemouth and Poole. So now that didn’t play out as a dynamic amongst the [hospital] collaborative because we were different types of providers and we weren’t competing for patients. So [Provider 1] being cardiac, lung specialists, [Provider 2] being cancer, [Provider 3] being general, we’d already kind of got through.“* (03; Director of Improvement; Hospital Group 2)

#### Skirting competition requirements

A common theme in the interviews, which was also acknowledged in the White Paper, was that many local leaders were implementing complex and overly bureaucratic governance structures which allowed them to navigate around rules introduced by the 2012 Health and Social Care Act. For example, one Leader noted, in relation to their association of provider trusts, that “*we were getting all of the benefits of what you’d get through [a merger] without having to spend two years distracting everyone on a disruptive merger process*” *(18; CEO; Hospital Group 3)*. This indicates that, as the White Paper outlines, organisations were operating upon informal collaborative arrangements to avoid legal and anticompetition mechanisms. In some cases, these rules were an annoying quagmire to navigate, but in others, the rules were just a challenge in creativity to overcome:

“*Yeah, I mean, I think the whole cultural backdrop in the NHS is radically different now isn’t it so, you know, we’ve still got the 2012 Act but none of us really adhere to the spirit of it so I think, you know, that in itself has kind of created the climate for collaboration hasn’t it*.” (06; Provider Association)

One policymaker agreed that the drive for competition brought about by the 2012 act was becoming outweighed by the benefits of collaboration, and the benefits of a market system were not being realised:

*“My sense is that over the last three or four years the need for collaboration is outweighing any benefits that we think that we get from a competitive marketplace.”* (04; Provider Policy Leader)

#### The impact of competitive commissioning on collaboration

The proposals moving commissioning functions of CCGs and parts of those of NHS England into the ICS to enable a more collaborative and system-focused commissioning model were captured within the interviews. The current CCG workings were outlined in our findings as a barrier to collaboration and system-level working. A general lack of trust between CCG and provider was a common theme. One NHS Leader intimated that they perceive that the local CCG does not trust them, and therefore they do not trust the CCG either, and that this hampered efforts to engage in a potential merger:

*“They don’t trust where the money’s going, they don’t understand [ … ] if we agree to this merger we’re not sure that the pound’s going to go where the pound should go and the right services will be provided for residents so it was a lack of faith in the CCG.”* (15; Chief of patient rep. body; Range)

Although competitive tendering is only formally required in a small number of cases, one high-level provider executive acknowledged that some CCGs made providers tender annually for services. This fostered a climate of short-term thinking, where:

*“ … you live on a kind of annual basis, the real impact of competition which is basically that your local CCG will put out your services to tender every year if it fancies it”* (11; NHS Provider Association)*.*

This provides evidence that some CCGs may rely on this overly restrictive process, hampering cooperation and may limit desire to engage in any kind of long-term investment in collaborative arrangements that could potentially benefit the local system.

Additionally, with the movement of the CCGs into the ICS body (and a historical context of the number of CCGs being continually reduced), the interviewees understood that the providers are going to increasingly become their own “referees” in the absence of competition regulation. As such, potentially, a kind of “global budgeting system” would be implemented, whereby:

“ … *all of the people who are involved in transacting commissioning are then immediately not required.”* (04; Provider Policy Leader; Range).

### Improving accountability

Interviewees acknowledged that the current system often resulted in weak lines of accountability. The lack of statutory underpinning for ICSs, for example, made accountability more opaque. One NHS Leader suggested that the 2012 reforms led to there being “*almost so much accountability everywhere that true accountability gets lost in that system”.* The lack of statutory basis for ICS’ led to there being no legal basis for the leader to be on the ICS board, further undermining accountability and trust:

*“And the irony is that although there’s one [ … ] ICS, it doesn’t exist as an entity because there’s no legal foundation for it to exist, there’s nothing in the legislation which allows for the existence of that and that’s where we need to be”* (15; Chief of patient rep. body; Range).

#### Inspections and care quality commission (CQC)

We found that NHS leaders were generally sanguine with regard to the current role of the CQC. However, there were also concerns about the new role for the CQC in moving to ranking ICSs and performance on a system level and how these aspects will coexist with organisational accountability [37]. For example, there are concerns that one organisation will be disadvantaged with the award of a poor rating for aspects of performance that another organisation is responsible for:

*“So, we kind of have to go to our system to be held to account for administrative failures within the Trust, that are really nothing to do with our partner colleagues across the system. But, if we failed to do that, then will they be tarred with the same brush?”* (02; Director; Hospital Group 1)

This illustrates that there is a danger that the new rating systems may undermine, rather than foster, collaboration. Policymakers also suggested that they try not to get involved with decision-making around mergers or other collaborative activities, but they do try to reflect on the quality of the organisations involved. However, one NHS Leader put forward the view that they did not feel that the way in which they were judged in inspections took the local context into account and that some degree of reform is required:

*“Trusts say that is a really big barrier to collaborating because in a way they’re kind of still being measured on the performance of what they do within the four walls of their trust and much less so on what they’re doing as part of their system [ … ]. So, I think that’s an area where we would encourage regulators to kind of take a slightly different view of, you know, what is performance in a system and how do you measure that? And then, there’s a wider question of, you know, ‘does CQC have their remit to performance manage a system, regulate a system?’”* (06; Provider Association).

### Enhancing external control over local health systems

Increased centralisation of power away from local systems towards the national government presents risk of greater ‘interference’ in how these systems are run and would require trust in the government to only intervene in a beneficial manner. However, one of the major themes identified in the interviews was that even the current regulatory arrangements were eroding trust between organisations and that this lack of trust has also historically manifested vertically in the system.

#### Lack of trust in the commissioning and regulatory systems

Providers had little faith that NHS England/Improvement would deliver funding that was previously promised to implement collaborative arrangements. A leader reported that they were promised funds from NHS E/I to implement an arrangement with the aim of taking over another organisation to improve its performance, but that these funds were not forthcoming:

*“ … and in the end, we weren’t supported financially in the way that I was led to believe we were going to be. And so we had a choice about whether we stayed doing what we were doing, or withdrew [ … ] we decided that we would stay, but [Interviewee’s Trust] suffered as a consequence of that, because it had to divert its own resources and people into [Other Trust], and we weren’t given the financial support to do that.”* (12; Former CEO; Alliance 1)

A Leader, too, reported that financial promises by regulators were not kept:

*“There was some early money dropped in, it was about £28 million was dropped in. And we were promised significant funds each year thereafter [ … ] we got £28 million, nothing else came [ … ] it created a lot of bad feeling, and we were deep into the improvement journey then, and felt we couldn’t … we had a lot of discussion around, “Should we step away?” [ … ] the other things that we wanted to do, we’ve just not been able to.”* (10; Exec Nurse; Alliance 1)

It is clear that broken financial promises caused a significant erosion of trust between providers and regulators. This manifests in a loss of faith in the system and leads to NHS leaders not wanting to undertake further collaborative efforts. Without additional financial resource, taking over a lower preforming organisation causes a loss in performance in the higher performing one. This often makes these types of collaboration unattractive for NHS leaders.

#### Exertion of external control and its impact

The lack of trust in regulators was also reflected in other ways, in some cases, with regulators intervening by demanding changes to how ICSs operated. It was evident that there was some ability for local leaders to negotiate with regulators about these changes. But there was nonetheless a perception that regulators were less well-informed than local leaders about what specific local changes would be most helpful. As one leader of an ICS noted, they are not happy with enforced changes to how a successful ICS is already run:

*“At the 11th hour, the government department decided that we should insert controls, so I just said no, I ain’t doing that, because it would just undermine every single thing we have done, and show they are actually not really interested.”* (13; CEO; ICS 1)

These concerns present issues for the proposed increase in power of the government in decisions previously made by NHS England. Leaders also highlighted problems associated with having to navigate public perception of privatisation in the light of the 2012 Act, which dampened their enthusiasm for engaging in collaborative arrangements linked (accurately or not) to privatisation:

*“We went out to the market to find out whether we could put an MCP [Multispecialty Community Provider] type organisation, if there was any interest. That was done over the winter of 2017 against the backdrop of the judicial review as well, saying this was the privatisation of the NHS in the northeast [ … ].”* (16; Manager; Integrated Care Provider).

This demonstrated that there were fears that government initiatives were linked to privatisation, which undermined genuine attempts at collaboration if not ‘marketed’ correctly.

### Data sharing across the system

The White Paper also makes important reference to improving data sharing across the system. In the Health and Care Bill the main aim is claimed to be “﻿*to enable the Department of Health and Social Care and NHS England to publish mandatory information standards to ensure providers of health and adult social care adopt a standardised approach to the collection and processing of data*” [16].

The ability to share data across organisational boundaries is, of course, essential to the process of inter-organisational collaboration. Interviewees generally espoused that the NHS was very behind in this respect:

“*What we are rubbish at... We say that we’re good at exchanging information between Trusts, we’re not, we’re rubbish at it; no one wants to admit if something’s not working.“* (03; Director; Hospital Group 2)

Lagging behind in this way raises issues about their ability to collaborate, as it can not only negatively impact patient care and the sharing of patient information, but also the sharing of staff information can undermine trust between organisations:

*“So, lots of the background work that goes on in managing and running the service is obviously reliant on HR processes, on workforce teams supporting how we plan our rosters and how we hold our staff data; and those things are still organisationally dependent so even though we’ve come together in October as [Merged Entity] you’ve still got terms and conditions dependent on where you were employed. And I think those things create animosity between staff members but also huge practical barriers”* (21; Director; Merger)

However, data sharing is not only essential to enabling collaboration, but also to improve patient outcomes. Sharing of patient data may also rely upon a degree of inter-organisational trust, particularly when working cross-sector. When combined with the above, this creates a catch 22 situation, where you need trust to share data, and data is needed for ability to collaborate. How the legislation might address this is not clear:

“*I think there's a view that it's really hard to get everyone from primary care to sign up to data sharing agreements. We've managed to do it locally after a lot of blood, sweat and tears, and trust, because you need the trust*” (16; Manager; ICP North)

Being able to share data is one part of the puzzle, but the next hurdle is to understand how best to utilise these data to improve patient outcomes. While the new legislation may enhance data interoperability, it is not clear how it will provide guidance on the best use of that data:

*“I would say that the digital challenge and then using that intelligence to be able to drive change is the next bit. It’s the ‘so what’. You're getting your data, yeah, that's lovely, what does that mean? What are we going to do with that information?”* (16; Manager; ICP North)

However, interviewees noted that changes during the pandemic had improved the ability to share data and has resulting in specific data sharing initiatives, such as the NHS COVID-19 data store [38]:

*“ … you can’t use the sort of information I suppose on the back of Covid, we know a lot of that stuff, we’ve been sharing our data, you know, people have, as I understand it, have now got like system wide waiting lists which they haven’t before partly because all of that was put on hold”* (09; Policy Transformation Lead)

While improving data sharing is seen as key to the success of future collaborative arrangements, publishing data standards may be the minimum that can be done to foster these developments. How these data are used, and how one may encourage unwilling organisations to share these data, are an open question.

### Inter-organisational collaboration during the COVID-19 pandemic

The DHSC White Paper explicitly mentions the pandemic and states that “*we must not go back to the old ways of working. The gains made through these new approaches must be locked in*” [17].

#### Changes to improve collaborative efforts during the pandemic

The COVID-19 pandemic had seen some “red tape” that applied in normal times lifted and standards relaxed in order to enable the large influx of patients to be treated in difficult circumstances. For example:

*“The CQC agreed to temporarily roll back a lot of the usual documentation that was done on patients in line with the Royal College of Nursing and other professional regulators to say, actually, you don't necessarily need to be doing this to keep patients safe.”* (02; Director; Hospital Group 1)

Additionally, one interviewee highlighted how suspension of fines for delayed transfer of care (DTOC) was another aspect that enabled increased partnership working:

*“ … [during Covid-19] so many people have just been in favour of the removal of bureaucracy so removing a ton of barriers to partnership working and to relationships has just been so helpful so whether that’s DTOC fines or continuing healthcare. I haven’t heard anybody sort of raise any concern or any anxiety about all of those bureaucratic burdens which were lifted and seem to have really accelerated kind of partnership working”* (06; Provider Association).

Likewise, it enabled pools of money to be accessed that didn’t exist before, freeing up capacity for collaboration across sectors:

*“ … all financial barriers have been removed, there was a central pool of money that the CCG, the medical authority can draw down from to work together to get people out, to get people home first and wrap that care around them at home”* (28; Local Government Representative).

#### Pressure as a catalyst for collaboration

The immense pressure on the health system drove key actors to seek resources outside of traditional organisational boundaries. This increased collaboration taking place in the context of COVID-19 was reflected by practitioners:

*“So that was the catalyst and the trigger for, I guess, networks to develop, informal networks of leaders, between organisations. So, people who traditionally hadn’t necessarily picked up the phone to each other, to just … what I would call the mutual aid agenda. So how can we help each other”* (04; Provider Policy Leader)

Similarly, the practitioners acknowledged that the shared trauma of the pandemic enhanced their ability to make close interpersonal connections across organisational boundaries*: “So people formed bonds of trust over a really short period of time, just because of the intensity of what was going on”* (04; Provider Policy Leader). However, there was also significant concern about what might happen when temporary regulatory allowances are lifted and a return to ‘normality’ occurs in the absence of regulatory reform. This is summed up by the following quote by an interviewee:

*“COVID’s been a great catalyst for collaboration in a lot of places and it’s a great example of what you can see happen when you put a shared purpose in, a shared goal in place in a system and then you take away all the barriers to collaborating to meet that shared purpose. How that will then work in the real world when COVID kind of recedes and all the regulatory barriers get put back in place - god forbid”* (06; Provider Association).

Another director of a hospital group echoed these concerns, highlighting that the second wave of the pandemic in 2020 was the impetus to drive greater collaboration in a time of great need, but that it might lead to regulatory growing pains to try to enshrine these developments in policy:

*“The second wave of COVID-19, things were much more extreme where we were. So we did manage to change the models of care. And then the legislation is trying to, I think, put walls around some of those things. They wouldn't necessarily had been there in normal time. So there'll be some difficulties in trying to mainstream that”* (02; Director; Hospital Group 1).

However, one interviewee highlighted the need for greater consultation between policymakers and regulators regarding the changes required to build upon the greater collaboration exhibited during the COVID-19 pandemic, warning that the changes made have not been sufficiently tested:

*“The provider collaborations that have actually sprung up from COVID-19 in particular have been legion and really interesting actually but at the moment there is a massive mismatch and I think, I think there has been a lack of desire from NHS England mainly and NHS Improvement and others to have a real conversation about what we want, they’ve just wanted to push through an approach and basically without, you know, we’ve got real incremental policy change without understanding the cumulative impact of all of these changes”* (11; Policy Executive).

## Discussion

This is among only a small number of academic studies that have interviewed NHS leaders and stakeholders with regard to the impact of legislative barriers collaboration and integration [3, 8, 39, 40]. Our findings highlight several important themes, not least how the requirement to compete has undermined collaboration, the historical bureaucratic burden presenting additional complexity to overcome, how NHS leaders are ignoring inappropriate regulations and seeking to work around these, the impact of CQC and inspections, overregulation, and the COVID-19 pandemic. The findings are also an important historical record of how a global pandemic changed the nature of collaboration in a health system which, while moving away from competition, still retained a competitive ethos and culture.

Both our findings and the DHSC White Paper indicate that the primary barrier to collaboration was the existing market system, the requirement to compete in the context of CMA regulation, and associated bureaucracy. It was clear that collaborative activity being blocked, such as in the case of the Bournemouth and Poole merger in 2013, as well as the substantial cost of going through the CMA approval process, were acting as mental barriers to collaboration in the minds of key actors [33, 41]. These findings were also reflected by Sanderson, Allen and Osipovic (2017), who stated that, after this highly publicised prohibition of a merger, there was a “﻿*move to avoid the escalation of merger proposals to the national competition regulators wherever possible*”. However, they also highlight that since then, all mergers have been allowed, given a more involved approach by what was then Monitor with Foundation Trusts. Nonetheless, our findings indicate that the uncertainty and cost the CMA created is still felt in the UK healthcare system as late as 2021.

Particularly noteworthy in our findings is the impact of ‘broken promises’ by regulators and CCGs on system-wide trust, where we found that lapses in financial commitments made resulted in a lack of desire to engage in future collaborative efforts. This suggests that subsummation of CCGs into the new ICS Body may bode well for more collaborative decision-making. Similar findings have been highlighted by Osipovic et al. (2016), who, in their investigation of competition versus cooperation in commissioning in the NHS, identified that “﻿*the existence of competition as a potential commissioning tool has decreased the amount of generalized trust between actors and made different NHS organizations more self-interested*” [39]. This emerged as some NHS providers, more than others, were prepared to lean on competition as a tool where it was advantageous to them, and in other cases, would cooperate more, emblematic of a system built upon ‘coopetition’. Yet, these competitive forces created mistrust between commissioners and providers in the system, as reflected in our findings. This historical lack of trust has also been recognised by Exworthy, Powell and Mohan (1999), who state that ﻿it could be argued that “c*onservative reforms changed the NHS from an organization based on trust to one based on contract*”. The proposed shift of power away from local systems towards Whitehall, perceived by some as a ‘power grab’, may further erode trust in the system [42]. Our findings, alongside others, indicate that NHS leaders feel that the locus of power should be shifting more and more locally rather than centrally [23, 42].

Others have explored what regulatory reform stakeholders in the NHS may prefer. A report by NHS Confederation, published in 2020, interviewing NHS leaders, identified that ICSs should be given a statutory underpinning, that novel regulation should incentivise greater joint working (similar to the new duty to collaborate), and that the locus of accountability should be moved into the ICS to focus on local priorities [43]. These findings broadly align with what we have identified in our study and provide support for the proposed changes in the White Paper. A report by NHS Employers investigated case studies of Vanguard arrangements [44] and focused on trust between organisations, outlining a number of means by which regulation affected the ability for organisations to collaborate effectively [44]. Key to their findings was the perceived unpredictability of the regulators, which we also identified. However, we also found a lack of trust between providers and commissioners, which made it difficult for providers to engage in long-term planning. A report by NHS Employers (2017) which conducted interviews during the Vanguard programme also highlighted the lack of trust between providers and regulators, with one individual they interviewed stating, “*What really will the Department of Health do, or will NHS England really support us*?” [44].

Despite our analysis suggesting that the change in policy by the DHSC is a useful step towards removing barriers to collaboration identified by stakeholders, it is unclear whether greater collaboration and integration alone will be enough to improve health system performance. Hudson (2021) notes that much of the market bureaucracy will still be in force in the NHS’ main partner - adult social care, and that there is potential for market bureaucracy to simply be replaced by state bureaucracy, with accountability moved ‘upwards’ [45]. Additionally, our interviewees highlighted that being told *how* to collaborate within current arrangements that do not perfectly align with the new mandate represents an increase, rather than reduction in, bureaucracy, and that such changes may undermine nascent interpersonal and inter-organisational relationships. Similarly, Alderwick et al. (2021) draws attention to the fact that the NHS has been undergoing almost constant reorganisation for the past 30 years, and that these reorganisations have apparently delivered little benefit [46]. The requirement to constantly reorganise can drain resources and staff confidence. Instead, policies that improve investment, expand the workforce, and modernise services, may produce better outcomes [46]. Likewise, continual reorganisation, regardless of direction, has also been found to discourage partnership working [47].

With respect to the pandemic, NHS leaders, policymakers, and patients all recognised its importance in helping to foster collaboration over competition, but they were also wary of a return to the ‘status quo’. There was a consensus that the barriers removed during the pandemic should not be reinstated. Therefore, and similar to others [22, 46], our findings indicate that enacting legislative change in the wake of COVID-19 should be done cautiously and with respect for the structure of existing collaborative arrangements. While interviewees espoused how COVID-19 improved attitudes towards collaboration while also removing significant red tape, capacity for system-wide reorganisation is lacking.

### Recommendations for policy and practice

While the 2021–2 Health and Social Care Bill offers a step in the right direction, there is much still to be done to address the deficiencies in social care, workforce, and to nurture genuine trusting relationships both vertically and horizontally in the health system [16, 42]. Policymakers and regulators need to be mindful not to disrupt the existing balance of established relationships and structures that have been carefully negotiated during the difficult context of the 2012 Health and Social Care Act by expecting drastic improvements from these new measures quickly [20]. While the Bill in many ways simply brings the law up to date with what has already been going on locally within the system, it also brings in new ways of working. Some latitude should be given to those systems which need time to reconfigure their existing ways of working to the new ones. Policymakers should also provide clarity or implement safeguards regarding when the Secretary of State’s new powers can be used to overrule localised decision-making, as this has the potential to undermine the place-focused healthcare that the Bill itself espouses. If this is not the case, as our findings demonstrate, such incidents can have long-lasting impacts [48].

Similarly, while the ‘hardware’ such as legislation and the formal structure of collaborative organisational entities are important, they rely upon the ‘software’ of interpersonal and interorganisational relationships to be properly implemented. Key actors working in local health systems that are new to collaboration, or had not worked as part of an ICS before, should endeavour to approach these changes with an open mind and look to recent guidance on how to build a collaborative mindset within their organisation and with their partners [49].

With specific regard to inspections by the CQC, NHS Employers (2017) found that inspections could cause partners involved in collaborating to turn inwards and revert to negative self-interested behaviours. This reinforces the need to include some reform to the way CQC handles inspections in collaborative organisations. Although the White Paper did not explicitly state how ICSs would be rated, the government has since stated that the CQC will have a similar role with ICSs as it currently does with hospitals [37]. Our findings suggest that rating collaborative systems requires a different approach than that currently taken with single providers, perhaps ensuring that collaborative behaviour itself is a component of what is assessed. Such a system could draw on findings from the present study and others relating to how functioning of inter-organisational and cross-sector collaborations can be optimised [27, 49, 50]. The concern, according to our interviewees, is that these new rating systems may unintentionally penalise collaboration, particularly in situations where partners may be ‘dragged down’ by poorly performing collaborators. This landscape must be navigated carefully to avoid making key actors fear collaboration. As such, the new ICS-focused rating system should ensure to consider that organisations may have to reduce their individual performance in order to help out others in their local health system. Such altruistic organisational behaviour should be rewarded and not penalised. Likewise, the focus of rating systems and inspections should also not initially be on demanding specific structural reforms during a time in which the focus should instead be on reducing the unprecedented elective surgery backlogs and improving staff shortages, in a context of insufficient financial resource.

Lastly, our findings show that current inability to share data presents barriers both to workforces working together as well as to leveraging it to improve patient care. Others have identified further barriers to data sharing in the NHS to foster collaboration, where standardisation is only one of them [51]. These barriers include the presence of external incentives for clinicians to engage with quality improvement built upon these data, and ensuring proper data collection [51]. Our findings show that data sharing is both essential to enable collaboration, and predicated upon it, creating an inherent ‘catch-22’ style dilemma. Creating data standards to improve interoperability between providers may help significantly to break down remaining barriers to collaboration.

### Limitations

There were several limitations to our current research. One is the small number of interviewees in sample, which was partially attributable to practical difficulties arising from the COVID-19 pandemic. We do not think we reached theoretical saturation and that there may have been more information and insights to uncover. For example, the White Paper also proposes reforms to the national tariff payment system, but these changes were not explored fully in our interviews. In terms of content of our analysis, it was strong in terms of the barriers presented by existing regulation but was relatively weak on what could be done to remove or surmount them.

## Conclusion

This paper provides novel analysis regarding barriers to integration and collaboration in the NHS in the immediate period before the release of the “Innovation and Integration” White Paper by the DHSC. We performed qualitative, realist interviews with a mixed “issue network” sample of 30 NHS leaders, commissioners, regulators, and policymakers. Our findings demonstrated that the majority of the DHSC legislative changes corresponded to barriers identified by stakeholders, particularly by removing requirements to compete, incorporating a duty to collaborate, reducing bureaucracy, and by clarifying accountability in local systems. Although COVID-19 led to the temporary removal of many barriers to collaboration, there was uncertainty among stakeholders regarding whether these would be re-implemented, which may undermine the motivation to collaborate in the future. However, we also identified a historical lack of trust between providers and regulators, which is unlikely to be addressed by this regulation. The proposed shift in power from NHS England to Whitehall may also erode trust in the wider health system. Finally, how the shift from competition towards collaboration will solve fundamental issues, such as staff shortages, is far from clear. We conclude with specific recommendations for policymakers and those implementing such arrangements, for example, highlighting that care must be taken to ensure that mandated changes to collaborative forms such as the introduction of the ICS NHS Body do not undermine the existing relationships formed in non-statutory inter-organisational collaborations.

## Supplementary Information

**Additional file 1.**

## Data Availability

The datasets generated and/or analysed during the current study are not publicly available due to the requirement for anonymisation of involved organisations and persons but are available from the corresponding author on reasonable request.

## Abbreviations

CCG: Clinical Commissioning Group
CMA: Competition and Markets Authority
CQC: Care Quality Commission
DHSC: Department of Health and Social Care
ICS: Integrated Care System
NHS: National Health Service
